# Introducing a MAP for adherence care in the paediatric cystic fibrosis clinic: a multiple methods implementation study

**DOI:** 10.1186/s12913-021-07373-5

**Published:** 2022-01-26

**Authors:** Bianca Richards, Sonya R. Osborne, Megan Simons

**Affiliations:** 1 Occupational Therapy Department, Queensland Children’s Hospital, Children’s Health Queensland Hospital and Health Service, 501 Stanley Street, South Brisbane, Queensland 4101 Australia; 2 School of Nursing and Midwifery, Faculty of Health, Engineering, and Sciences, Centre for Health Research, Institute for Resilient Regions, University of Southern Queensland, 11 Salisbury Road, Ipswich, Queensland 4305 Australia; 3 Australian Centre for Health Services Innovation (AusHSI), School of Public Health and Social Work, Queensland University of Technology, Victoria Park Rd, Kelvin Grove, Queensland 4059 Australia; 4 Centre for Children’s Burns and Trauma Research, The University of Queensland, Child Health Research Centre, 62 Graham Street, South Brisbane, Queensland 4101 Australia

**Keywords:** Cystic fibrosis, Adherence, Pediatrics, Implementation science

## Abstract

**Background:**

People with cystic fibrosis are required to adhere to a burdensome daily treatment regimen. Comprehensive adherence protocols can support more consistent use of adherence interventions and improve treatment adherence rates. This study aimed to explore the feasibility, acceptability, and appropriateness of implementing an adherence protocol into the outpatient cystic fibrosis clinic of a tertiary, paediatric hospital.

**Methods:**

This implementation study employed a pre-post observation design, using multiple methods. Focus groups and semi-structured interviews were conducted pre-implementation to understand clinician and consumer perspectives on adherence care. A multicomponent adherence protocol (including multidisciplinary written treatment plans, digital mental health screening and customised communication tools) was then implemented as standard care for a three-month implementation phase. Quantitative data was collected throughout using purpose-designed audit tools and surveys. The Replicating Effective Practice (REP) Framework guided the implementation process. Analysis was informed by The Consolidated Framework for Implementation Research (CFIR) to identify factors that support or challenge the integration of adherence protocols into standard care.

**Results:**

Thirteen clinicians, eight parents and two adolescents participated in focus groups or interviews that informed development of the tailored multicomponent adherence protocol for implementation. Medical chart audits demonstrated that the protocol was used with 44–57% of eligible consumers three months after introduction. Eighteen clinicians and five consumers participated in post-implementation phase questionnaires. The protocol was considered acceptable and appropriate to clinicians and consumers. Changes in clinicians’ practice behaviour were short-lived peaks in response to targeted intervention strategies throughout the implementation phase, such as audit and feedback.

**Conclusions:**

An adherence protocol is not an “off the shelf” solution to the adherence challenge in a hospital outpatient setting. Despite the tailored adherence protocol being considered appropriate and acceptable to clinicians and consumers, low fidelity indicates limited feasibility in the outpatient clinic setting, where multi-disciplinary members are all considered responsible for adherence care interventions. Key implementation factors and strategies to consider prior to introducing an adherence protocol are described.

**Trial registration:**

Australian New Zealand Clinical Trials Registry ACTRN12619001730190 (Retrospectively registered).

**Supplementary Information:**

The online version contains supplementary material available at 10.1186/s12913-021-07373-5.

## Background

Cystic Fibrosis (CF) is a genetic disorder of the exocrine system which affects the lungs and digestive system as well as pancreas, liver, kidneys, and intestines. People living with CF are required to complete a daily treatment regimen to manage their disease progression; typically including oral medications, nebulized medications, nutritional supplements, increased caloric intake and daily physiotherapy treatments [1]. This takes on average 100 min per day to complete [2].

The efficacy of modern CF management is dependent upon the patient’s adherence to their prescribed daily treatments [3]. Adherence is defined by the World Health Organization as “the extent to which a person’s behaviour – [that is], taking medication, following a diet, and/or executing lifestyle changes – corresponds with agreed recommendations from a health care provider “ ([4], pg. 18).

Across both paediatric and adult CF care, sub-optimal adherence is a concern. Data on adult adherence suggests that completion of inhaled therapies averages 36% of the agreed prescribed frequency [5]. Adherence varies according to the treatment type (nebulised medication vs enzymes) and reporting type (self-report vs objective data) [6–8]. Sub-optimal adherence has been linked to negative outcomes for both the individual and the health care system, including reduced baseline lung function, higher occurrence of pulmonary exacerbations, greater risk of hospitalisation, increased intravenous antibiotic usage and higher associated health care costs [7, 9].

The optimal interventions to increase patient adherence remain unclear, as reported in a recent meta-analysis into medication adherence interventions for self-administered medications [10]. Current clinical guidelines suggest the use of multi-component adherence interventions designed to harness the combined effectiveness of multiple intervention elements [11, 12]. Multi-component adherence interventions may integrate elements of psychoeducation, formal adherence assessment, clinician communication skill training and behavioural approaches (such as exploring beliefs, counselling and collaborative decision making), as well as organisation changes (such as educating training care teams, utilizing alternate care models) [11, 13, 14].

Few multi-component CF-specific adherence protocols focusing on clinician behaviour have been described or evaluated in the published literature. One such protocol is IMPACT [15]. The IMPACT protocol, designed for young adults (11–20 years old) with CF, is a combined adherence assessment and intervention protocol [15]. It combines elements of educational, organisational and behavioural adherence approaches, packaged as a set of tools for use in a CF outpatient clinic environment [12]. When trialled across 18 CF health care centres in the United States, no significant treatment effect was recorded on the key outcome measure of medication adherence [12]. However, the research team reported systemic barriers in the process of implementing the IMPACT protocol (clinical demands, clinic space constraints, limited time to conduct the intervention and low clinician attendance at supervision and training sessions) [12]. These ‘on the ground’ barriers echo previously published adherence projects where the challenges at a health care level impact upon the ability of adherence projects to transition from ideas to integrated practice [13, 15–17].

Understanding and addressing the factors impacting on effective implementation of an adherence protocol can support clinical teams to integrate sustainable adherence interventions into their daily work. The emergence and growth of the field of implementation science provides researchers and clinicians with a theoretically or conceptually derived systematic approach to identify factors likely to inhibit or enable successful translation and implementation of evidence-based interventions. Implementation science has been defined as “the scientific study of methods to promote the systematic uptake of research findings and other evidence-based practices into routine practice, and, hence, to improve the quality and effectiveness of health services” ([18], pg 1).

The aim of this study was to explore the feasibility, acceptability, and appropriateness of implementing a CF treatment regimen adherence protocol (herein termed ‘Multicomponent Adherence Protocol’ [MAP]) into an outpatient CF clinic in a tertiary paediatric healthcare setting.

## Methods

### Study design

A pre-post observational study design, using multiple methods was employed. Quantitative data was collected to evaluate service use, delivery of, and staff fidelity to the MAP components. Use of multiple methods allowed rich qualitative data to be collected to explore factors likely to influence the implementation and hence tailor the adherence protocol for program sustainability. The Replicating Effective Programs (REP) framework [19] was selected to inform the implementation of the MAP.

The study was approved by the relevant hospital (HREC/18/QCHQ/44458) and university (2,018,002,220/HREC/18/QCHQ/44458, 1,800,001,158/ HREC/18/QCHQ/44458) human ethics review committees (HREC) and will be reported following the Standards for Reporting Implementation Studies (STaRI) guidelines [20].

### Study setting

The study was conducted in the outpatient CF Clinic of a publicly funded, tertiary-level, teaching hospital located in South East Queensland, Australia. This clinic is the primary care provider for all children and young people diagnosed with CF (0–18 years of age) across the state of Queensland and northern New South Wales in Australia, with approximately 350 patients at the time of the study. The CF clinic team is multidisciplinary and is comprised of a core group of respiratory physicians, CF specialist nurses, senior allied health professionals (Physiotherapy, Occupational Therapy, Social Workers) and a rotational group of dietitians, physiotherapists, social workers and occupational therapists.

### Participants

Participants in the study were selected using purposive sampling methods to represent the key stakeholders in the study clinic; young people (aged 8–18), their parents/ carers and the multi-disciplinary team clinicians.

#### For young people with CF and their caregivers

##### Inclusion criteria

- A young person with a confirmed diagnosis of CF receiving care at the study clinic
- Parents/ carers of child or young person with a confirmed diagnosis of CF who receives care at the study clinic

##### Exclusion criteria

- Young person receiving care at the study site as an inpatient.
- Young person receiving care by the CF clinical team off site (i.e., hospital in the home).

#### For clinicians

##### Inclusion criteria

- All clinicians providing direct care to patients at the study site clinic during the study period (CF clinical nurses, respiratory physicians, dieticians, occupational therapists, physiotherapists and social workers).

##### Exclusion criteria

- Student trainees of any health discipline
- Respiratory Scientists
- Psychologists (as psychology is not available as standard care in the study setting)

### Recruitment and consent

Recruitment occurred between September 2018 to July 2019. The principal investigator (BR) was a member of the clinical team at the time of the study. The research assistant was familiar with the paediatric hospital setting but had not been involved with the CF outpatient clinic.

#### Pre - implementation

All clinicians in the CF team were invited by the principal investigator to participate in focus groups via the existing weekly team meeting and email. A sample was sought that included senior leaders and clinical staff, members of each allied health/ medical profession and clinicians with variable levels of experience. The number of anticipated participants for focus groups was 10–12.

Young people (aged 8–18 years) and parents/carers (with children 0–18) were invited to participate in the interviews. Five to ten consumer participants were sought. Following demographic analysis of the clinic population, the research team used purposive sampling to capture a sample that represented the various age groups of the clinic. As such, at least two participants were sought to represent: children 0–5 (parents), children 6–11 years (parent), young adults 12–18+ (parent and young people). Eligible participants were approached at their CF clinic by a member of the clinical team (not involved in the research project) to introduce the study. If interested, the research assistant provided information on the study and obtained written informed consent.

All participants were provided with an information sheet outlining the purpose of the research and their rights and responsibilities when participating in the study. Clinicians, young people and parents/carers were informed that participation in the research was voluntary, any information provided would be confidential and that they could choose to withdraw at any time. When recruiting young people, their parent/carer was involved in co-consenting and consulted as to whether the young person was capable to participate either alone or with their parent/carer present. Where appropriate, with child and/or parent/carer consent, young people who participated were invited to be interviewed without a parent/carer present.

#### Post - implementation

Clinicians, young people and parent/carers were recruited to complete a post-implementation questionnaire. This was a convenience sample of staff and consumers available to participate during week 6 (midway) and week 12 (final) of the implementation phase. Participation in the pre-implementation phase was not required.

### Implementation plan

The REP framework [19] was used to support the implementation of the MAP into the CF clinic. This framework was designed to support translation of effective health service interventions (e.g., adherence protocol) into health care and focuses on key stakeholder engagement through all phases of implementation [19]. Embedding the CF adherence protocol into routine service delivery requires a change in attitude and behaviour on the part of the clinicians, as well as the young people and their families. Thus, the REP framework is an ideal implementation framework because it focuses on key stakeholder engagement through all phases of introducing the new model of service delivery. The implementation plan based on the REP phases is outlined in Table 1.

**Table 1:** Study design outline following the four-phase REP framework [19], including key implementation activities and data collection

Phases	Pre-Conditions	Pre-Implementation	Implementation	Maintenance and Evolution
Timeframe	3 months (October to December)	3.5 months (January to mid-April)	3 months (mid-April to July)	1 month (July)
Activities	Conduct local needs assessment via consumer interviews and clinician focus groups Assess for readiness and identify barriers and facilitators	Facilitation: Collaborate with local clinicians to integrate tools into clinic structures and processes. Revise professional roles Develop a formal implementation blueprint Conduct educational meetings and distribute educational materials	Trial the MAP intervention Conduct ongoing training Facilitation: Collaborate with team members to understand implementation barriers and changes indicated. Monthly Audit and Feedback to clinical team	Evaluation via: clinician surveys Parent surveys Create resources to support sustainability, scale up and spread.

Implementation intervention activities have been named in accordance with the taxonomy of implementation strategies described by Powell et al. [21] which reflect definitions compiled by a panel of 71 implementation and clinical experts through a systematic consensus development process.

### Standard care

Prior to this study, standard adherence assessment was typically completed by any/ all members of the clinical team at each clinic appointment with results recorded in the electronic medical record. Parents and staff discussed that assessment was occasionally repetitive across multiple team members. Adherence interventions included provision of education, goal setting, encouragement, coaching, creation of reminder systems, providing options to modify how treatments are completed and supporting families to create daily treatment routines. All members of the CF multidisciplinary team had some involvement in adherence assessment or intervention: CF nurses, CF respiratory physicians, occupational therapists, physiotherapists, dieticians and social workers. Psychology support could be requested from an external team for more complex or targeted adherence care above what was considered standard care. Adherence assessment and intervention sessions occurred within the CF outpatient clinic, via home visits and in additional outpatient appointments booked outside of the clinic. These additional visits were conducted via face-to-face, telephone, email and/or telehealth platforms. The frequency of appointments ranged from three monthly to weekly reviews.

Wide variation existed in the clinical services provided between families at the study site. There was no clear timeframe or guidelines to define what interventions were offered or which clinicians were responsible for each intervention.

### Intervention – the MAP

In accordance with the REP framework [19], in the pre-conditions phase, a local needs assessment was conducted. Once an understanding of the context was achieved, the intervention (MAP, Table 2) was co-designed with members of the clinical team. This intervention was based on the original IMPACT protocol but adapted to fit the needs of the local setting [15]. The process of creating the MAP is further described in Results.

**Table 2:** Content of the Multicomponent Adherence Protocol

MAP components	Source	Definition	Frequency of use
1. Written Treatment plan	IMPACT Protocol [15]	A personalised written treatment plan that includes the patient’s treatment regimen, exacerbation plan.	Annually
2. Knowledge Assessment	IMPACT Protocol [15]	A multichoice questionnaire (Knowledge of Disease Management – CF) that assesses CF specific knowledge and understanding of treatments.	Annually
3. Emotional Wellbeing Screening	International Committee on Mental Health in Cystic Fibrosis [26]	A digital screening of anxiety and depression symptoms in all young people > 12 and parents. Utilising CF Mental Health Guidelines [26]. Screening assessments were administered via iPad using the Research Electronic Capture (REDCap) online surveys and database system	Annually
4. Clinic Communication Tool	New component	A document created for this trial. The one-page door sign was used by the clinic nurse/s in partnership with consumers as they accessed the clinic. Using this tool, families were informed of which clinicians were planning to review them and were able to request reviews as required.	Every visit
5. Problem Solving Intervention *Included but not evaluated in this study*	IMPACT Protocol [15]	Intervention program where clinicians collaboratively identify key barriers to adherence, and jointly problem-solve ideas with the young person.	Not included in this study due to availability of training materials and phased implementation design.
**Additional Components from IMPACT study that were considered in protocol design but excluded following local needs assessment.**			
Treatment Skills Assessment	Not included	Checklist based assessment. Outlines a list of treatment skills that can be observed to evaluate the young person/ parent’s ability in carrying out the plan.	Not included as this was already included as standard care at study site.
Adherence Assessment	Not Included	Treatment Adherence Questionnaire (TAQ-CF). A self-report adherence assessment tool, reviewing frequency and duration of treatment completion in the last 7 days.	Not included due to needs assessment results.

The MAP was piloted across the outpatient CF clinic for three months during the implementation phase. All families who accessed the clinic during the trial period received the MAP as standard care.

### Clinician training

All clinicians (*N* = 61) working within the clinic were informed of the MAP one week prior to implementation, when a two-page educational handout was circulated to the entire clinical team via email. In the first two weeks of implementation, three education presentations about the MAP were completed with members of the clinical team in attendance at existing meetings. In addition, a researcher was present for 60 min during four outpatient CF clinics to provide individual face to face training and problem solving to support implementation of the MAP. Feedback arising from audit data was provided monthly by the principal investigator (BR) via the existing clinical team meeting.

### Outcomes

The outcomes of interest in this study are the conceptually distinct implementation outcomes of feasibility, appropriateness and acceptability [18]. For conceptual clarity we contextualised the outcomes for the purposes of this study as follows:

- *Feasibility* is defined as the extent to which an innovation can be successfully used or carried out within a given setting [18]. As an aspect of feasibility, we also measured fidelity. Fidelity is the degree to which an innovation was implemented by the clinicians as it was intended by the program developers [18]. Fidelity was evaluated across three areas: adherence to the MAP, dose of program delivered, and quality of program delivery [18].
- *Appropriateness* is the perceived fit, relevance, or compatibility of the innovation for a given place, provider, or patient [18]. Appropriateness evaluates the observed fit between the adherence protocol and the clinic setting. Measuring appropriateness is valuable for picking up resistance or “push-back” to implementation efforts, particularly if the innovation is seen to be inconsistent with the organisation’s mission or the individual clinicians’ skills, role, or expectation for their job [18].
- *Acceptability* is the perception among key stakeholders that an innovation is agreeable and satisfactory [18] and acceptability outcomes allow the measure of perceived success of the innovation. Acceptability was based on individual clinicians’ and consumers’ knowledge of, or experience with, ongoing use of the adherence protocol and evaluated by assessing their level of satisfaction with various aspects of the protocol such as the content, complexity or comfort [18].

### Data collection and analysis

#### Clinician focus groups and consumer interviews

Clinician focus groups were facilitated by the principal investigator (BR) using a semi-structured question guide (Additional File 1). Their purpose was to explore clinicians’ existing adherence practices, perceived barriers and enablers to adherence work and clinicians’ readiness for change. The focus groups were audio-recorded and transcribed verbatim; field notes were also included in this data set.

Consumers were interviewed by the research assistant to explore how young people and carers/parents perceived adherence promoting interventions currently used in the clinic and determine the areas where change was indicated. The consumer interviews were conducted on site using a semi-structured interview guide (Additional File 2).

Thematic analysis [22] was completed on the clinician focus group and consumer interview transcripts. The purpose was to identify key contextual factors that should be considered prior to implementation and to determine the likely appropriateness and acceptability of an IMPACT protocol style adherence protocol [15]. Two rounds of coding were completed. The first round of coding was an ‘open coding’ round, where inductive, natural codes were identified in the transcripts. The second round of coding used the identified codes deductively by the Consolidated Framework for Implementation Research (CFIR) [23] to guide evaluation of the factors considered likely to influence implementation. The CFIR is a widely cited and rigorously developed determinants framework for implementation that was developed through a process of consolidating earlier published implementation literature [24]. The CFIR consists of five domains, containing 36 key constructs considered most prominent in influencing program implementation in terms of valence (positive or negative influence on implementation) and strength (strong or weak influence on implementation) [23].

All transcripts were independently coded by two coders (BR, MS). A third coder (SO) was consulted for reflexivity checking. Once CFIR factors were identified, two researchers (BR, MS) rated the predicted valence of each factor, with consensus achieved through discussion [25].

### Medical record auditing

Throughout the implementation phase, medical records of all patients who attended the CF clinic were audited to assess overall clinician fidelity to the MAP [21]. Audit and feedback were conducted as described by Ivers et al. [27]. Tailored checklists (Additional File 3) were used to compare the care provided with each component of the MAP.

Results were compiled monthly [27] and feedback was presented to the clinical team at an existing meeting by the principal investigator (BR). Fidelity was quantified as a percentage (i.e., number of protocol components completed and documented compared to the total number of applicable protocol components). A month-to-month comparison was presented visually and target goals and behaviours to improve implementation were identified by the principal investigator and key implementation clinicians for the following audit cycle [27].

### Technical assistance log

The technical assistance log was recorded by the principal investigator (BR). The log outlined all key implementation events and/ or external events that may have impacted the study, all formal and informal feedback received and any modifications to the MAP or implementation plan. The descriptive data from the technical assistance logs was reviewed at project completion alongside the quantitative and qualitative data to provide a narrative description of the implementation process and observe key activities that may have impacted implementation success. This data was compared with audit data to determine the overall feasibility of the MAP.

### Post - implementation questionnaires

Structured questionnaires using Likert scales and open comments were provided to clinicians, young people and parent/carer face-to-face at the CF clinic. Questionnaires were distributed at week six (halfway) and week 12 (final week) of the implementation period to assess acceptability and appropriateness of the adherence protocol. The questionnaires outlined each component of the protocol and asked questions related to how familiar the participant was with the tool and perceived value and fit.

Quantitative data was analysed using descriptive statistics. Clinician and consumer comments were analysed using thematic analysis and deductively applying the CFIR framework to determine key themes relating to acceptability and appropriateness using the protocol as standard care.

### Digital screening assessment

Digital screening assessments were introduced as part of the adherence protocol in response to concern from the clinical team that insufficient identification of psychosocial and mental health factors in their consumers was impacting upon treatment adherence. The tools (Patient Health Questionnaire-9 [PHQ-9] and Generalized Anxiety Disorder [GAD]) were selected and administered in accordance with the International Committee on Mental Health in Cystic Fibrosis guidelines [22] by the CF clinic social workers. The PHQ-9 and GAD are both freely accessible tools and are considered reliable and valid in this population group [22]. The tools were self-scoring and generated immediate feedback to clinicians to trigger a clinical response based on outlined care pathways (including referral to the Acute Response team, hospital psychology or community mental health supports). Throughout the study period, if children or parents’ responses triggered a suicidality flag on mental health screening (PHQ-9, item 9), immediate psychology review was arranged via the Acute Response Mental Health Team on site at the hospital.

Six months after the tools were introduced, descriptive statistics were completed on the data set to evaluate the total number of screens completed, the percentage of young people identified as “at risk” and the percentage who received follow up care, as part of fidelity outcome checking.

All findings arising from quantitative and qualitative sources were triangulated by the research team (BR, SO and MS) using the ‘following the thread’ methodology [28] to identify key factors and implications for future practice and research.

## Results

### Pre - implementation

Thirteen clinicians (92% female) participated in the three pre-implementation focus groups. Each group was comprised of three to six members of the clinical team and ran for an average of 45 min. All disciplines within the CF clinical team were represented (CF clinical nurse consultants (CNCs), nurse practitioners, physiotherapists, occupational therapists, respiratory physicians, social workers and dietitians). Eight caregivers (88% mothers) and two adolescents (50% female) completed the consumer interviews. The average interview length was 40 min.

### Factors impacting adherence assessment and intervention

Sixteen constructs of the CFIR Framework were ide ntified as themes within the interview and focus group transcripts. These constructs were identified as likely to either positively or negatively impact the introduction of a new adherence protocol into the clinic. Two additional themes, not included in the CFIR Framework, were identified: clinician-family relationships and parental decision making. All identified constructs and their predicted valence (pre-implementation) are outlined in Table 3.

**Table 3:** Pre-Implementation factors identified by clinicians, adolescents and parents/carers that impact adherence work

CFIR constructs	Factors identified by stakeholders	Predicted valence ^a^	Description/ Quote
**Inner Setting** **Structural Characteristics**	Social Architecture: Stability of Team	(−)	Multidisciplinary team with rotational allied health structure. Nursing team identified as most consistent by clinicians and parents and assume the coordinator role. *“Yeah, yes so we see everyone from OT, Social work, the nurse, doctor, physio” (Parent interview 1)* Instability of the team impacting consistency of care for families due to systems of communication, documentation and handover of adherence information. *“Clinic it’s harder because they [families] might be seeing a different therapist over all the different clinics and things like that get lost and don’t get passed on.” (Clinician focus group 3)*
**Inner Setting** **Structural Characteristics** **Networks and Communication**	Size of organization	(−)	Large cohort. Impact on time per family, team communication and planning. Large tertiary organization. *“I think another challenge is, because our clinic is so big, that our time as a team to get together to talk about patients is so limited, in a meaningful way.” (Clinician focus group 2)*
	Team relationships	(+)	Evidence of positive team collaboration on adherence work and recent focus on multi-disciplinary work. Team identified as ‘open and engaged’. *“…I know that over the past 6 months in particular, even 12 months, we have been trying to move towards adherence from an MDT (multi-disciplinary team) as opposed to individually within clinical areas”. (Clinician focus group 2)*
**Networks and Communication**	Team co-ordination	(−)	Clinicians perceived that adherence work was being completed by individual clinicians, within their scope of practice. However, they did not feel that this process was coordinated as a team. Perceived impacts included number of recommendations to families and work together on prioritizing goals. *“But I do think that as a whole, we are probably not integrating our adherence together, I think that we tend to still work very much on our own and on our own area that we work on.” (Focus group 2).* Both parents and clinicians discussed that clinic coordination resulted in longer, unpredictable appointments for families. Some parents acknowledged barriers around accessing the professionals they wish to see within their clinic appointment. *“Sometimes we need to talk to the [clinicians] or something about things, but they are often quite hard to get hold of. So, by the time they get hold of you, you’ve already resolved the issue coz you’ve talked to someone else or you just get over it and you just don’t want to talk about it anymore.” (Parent Interview 4*)
	Informal team communication	(−)	Communication between team members regarding adherence assessment or intervention was infrequent during, and outside of clinic. This resulted in reduced team awareness of adherence interventions underway with other clinicians and ensuring consistency of messaging to families. *“It’s hard especially in clinics, there’s not that communication with all the clinicians going in and out of what everybody is telling them [families] within that clinic. So, you don’t know how many things they’ve been given that day.” (Clinician focus group 2)* Parents also voiced concerns regarding team communication. “*It can be frustrating, very frustrating. It’s like is anyone, anyone on the same page? Like does anyone talk to anyone else?” (Parent 6).*
	Formal team communication	(−)	Team communication within formal communication structures such as meetings and clinical notes was reported to be challenging by the clinicians. Reduced clinician attendance and available time impacted the perceived effectiveness of communication in clinical meetings. Gaps were identified in clinician handover. Accessing adherence information in clinical notes was a barrier due to length of notes, available time in clinic for chart review and inconsistent systems in reporting adherence interventions. In effect, information sharing through the team was significantly impacted. *“The pre-clinic meeting should be a good opportunity to do that but sometimes I don’t feel like its necessarily as effective as it could be just because we are limited for time. Trying to run though all the patients and not everybody that’s at the meeting is always the one that’s been involved with the patient to really know the deeper level of information.” (Clinician focus group 2)*
**Culture**	Organisational culture “clinician flexibility”	(U)	Team discussions highlighted that clinicians had a high level of flexibility in how they conduct adherence work. This was guided by a culture where individualized care based on the perceived young person’s or family’s needs directs services provided, rather than outlined tasks or policies. *“… you have to be able to adapt what you do to the individual child and family circumstance.” (Clinician focus group 3)* *“I wouldn’t say that I have one particular goal, it’s just about trying to get the best outcome for them, however that looks for that family” (Clinician focus group 2)*
	Clinician beliefs “paternalism”	(U)	An underlying belief emerged within the clinician group that “adherence” is an unattainable target for families to achieve. Team members reported that they believed prescribed treatment plans are not realistic and place a large burden on families. As a result, goals and clinical decisions are influenced by this belief. *“It’s very easy when things are not going brilliantly with a kid to just keep adding in therapy. But you know, in a teenager who is busy and got school commitments and sport commitments and social commitments and let’s be realistic like… what are they actually going to achieve?” (Focus group 3)*
	Contrasting consumer beliefs to “paternalism”	(U)	In contrast, parents reported that they would prefer their team to discuss all treatments options and preferences with them rather than assuming family’s burden. *“I wonder, do they think that we already have enough? I’m just wondering, do they feel that “if we give them something else, are they not going to be able to manage” or something? I don’t know, there just seems to be hesitation in giving us more stuff. […]” (Parent 6)*
	Clinician beliefs “Adherence change is slow”	(U)	Beliefs about adherence work emerged. Clinicians discussed a shared belief that changing adherence is a slow process and that to see changes in adherence, a good therapeutic relationship with families is central. *“…sometimes we just have to plug away. Sometimes like dripping water on a stone, it might have some effect long term and we just have to keep doing what we are doing.” (Clinician focus group 2)* *“I mean generally speaking…The person that understands their disease less and feels that they have less of a relationship with their team and their consultant are not going to do as well.” (Clinician focus group 1)*
**Implementation Climate**	Receptivity to change	(+)	The team appeared open to change, perceiving “room for improvement” in standard adherence care. Clinicians were interested in innovations that were sustainable and supported timely delivery of adherence work. *“I think we could definitely improve on it [adherence work]” (Clinician focus group 3)* *“*… *And we could probably do it earlier. But I think we miss the boat a lot of times.” (Clinician focus group 3)*
	Available resources	(−)	Clinicians reported that time and staff resourcing impact current clinical care. No additional resourcing would be allocated to support implementation of an adherence protocol. “*I think clinic time is a big one for everyone. If we are all going to do really good, detailed, thorough education on every kid to help with adherence and the child’s understanding of the condition. We just don’t have enough time.” (Clinician focus group 1)*
**Readiness for intervention**	High awareness of user’s needs	(+)	Parents identified four key needs to improve CF clinic care: (1) need for increased social/ emotional support, (2) need for consistent team communication about treatments, (3) need for more efficient use of appointment time, (4) need for increased family involvement in treatment planning. All of these four key needs were independently identified by the clinicians who participated in the focus groups, suggesting that the needs of the CF clinic families are generally recognized by the organization. Both clinicians and parents identified that the clinic individualized the delivery of care to families. Relationships between families and the CF clinic team were considered high priority to both users and clinicians. Parents reported an overall positive experience of the CF clinic.
	Patient centred focus	(+)	*“Everybody just makes us feel... feel welcomed, as I said… doesn’t just treat us like just another patient (Parent 1)”*
**Outer Setting** **Needs and Resources of patients and families**	Individual knowledge and beliefs about adherence	(U)	Individual clinicians discussed that understanding of adherence impacts how adherence work is conducted. Adherence work was considered “hit and miss”. However, the reasons why sometimes therapy is effective and sometimes ineffective was not known to clinicians. Clinicians also expressed that adherence work can be challenging and clinicians can feel that their work is not impacting families. Multiple team members expressed interest in completing adherence work as part of their role. Parents reported that they believe the clinic has a role in supporting their adherence however, multiple parents could not identify a clinical intervention or aspect of CF clinic that directly impacts on home adherence. The parents reported that a commitment to “just get treatment done”, considerations about child’s best interests, family functioning and external support from the CF community were influential factors outside the clinic that influence home adherence. The majority of parents discussed that other people who have CF and/or their families are the best source of information to provide information about CF treatments.
**Characteristics of Individuals**	Impact of relationships	(U)	The relationship between families and the clinical team was discussed at length in both clinician’s focus groups and parent interviews. Maintaining a long-term therapeutic relationship was a key consideration of therapist interactions and considered central to affecting adherence. Parents discussed the positive impact of familiarity with the clinicians on the child and family’s interactions in the clinic, understanding the child’s preferences and supporting home adherence by referencing conversations and people known to the child when at home. *“You see I think like with adherence I really think that relationship building is so key and so if you can’t build that relationship because you don’t know that patient well or you don’t see them frequently enough it’s really hard to maintain that adherence.” – Clinician focus group 2)* *“I find that if I’ve known one of the staff longer, for a longer amount of time, I can talk to them easier.” – (Adolescent* 1)
Non CFIR constructs	Parental decision making	(U)	Outside of the interactions that take place in clinic, parents discussed how adherence at home is made more complex when they need to consider the “costs” of optimal treatment adherence at the family level. Parents of adolescents discussed that they had to rationalise and prioritise treatment recommendations in the context of their family unit, quality of life and relationship with their child with many families actively making sub-optimal treatment decisions to support family relationships and child’s quality of life. *“… it’s about my relationship with my kids. Coz I was really hard on my 17-year-old when she was going through a time of wanting more independence with her treatments and her health. And I just didn’t want to give that.... and we had a very, very poor relationship for about 12 months and that’s not worth it. Yep, I’d rather a good relationship with my kid.” (Parent*

### Feasibility and Fidelity

#### Adaptations to the IMPACT protocol

The original IMPACT protocol consists of five core components [12]. Four components of the original IMPACT protocol (Written Treatment Plan, CF knowledge assessment, Problem Solving Intervention and Treatment Skills Assessment) were deemed ‘a good fit’ for inclusion in the MAP (Table 2). Minor adaptions were made to the knowledge assessment and written treatment plan to support translation to the local site. The assessment of treatment skills was not included in the implementation plan as evidence suggested that its elements were embedded in existing clinic care.

The ‘problem-solving intervention’ was considered a central tenant of the adherence protocol by the research team however due to frequency of staff rotation in CF clinic, a digital education training package was created to support sustainability of the MAP. As such, the problem-solving training was not evaluated in this study.

Two non-IMPACT components (clinic communication tool and mental health screening tool) were added to the MAP to address identified gaps in coordination of care, communication and concerns from both parents and consumers about the impact of mental health on adherence in the study setting.

The ‘clinic communication tool’ was created to support team coordination and improve communication between the clinical team and families (Additional File 4). A digital ‘mental health screening tool’ was also introduced. This was a response to a perceived need from both families and clinicians for better support of mental health. By better identifying and supporting individuals in the clinic with their mental health, it was anticipated that more responsive adherence care could be provided and a major barrier to adherence could be identified early [23, 22].

The MAP was designed to be administered in full when patients attended their annual review clinic (a once-a-year appointment multi-disciplinary assessment and planning appointment). The clinic communication tool was rolled out for all patients attending the CF clinic, regardless of appointment type.

### Effectiveness of the implementation strategy

**Fig. 1:**
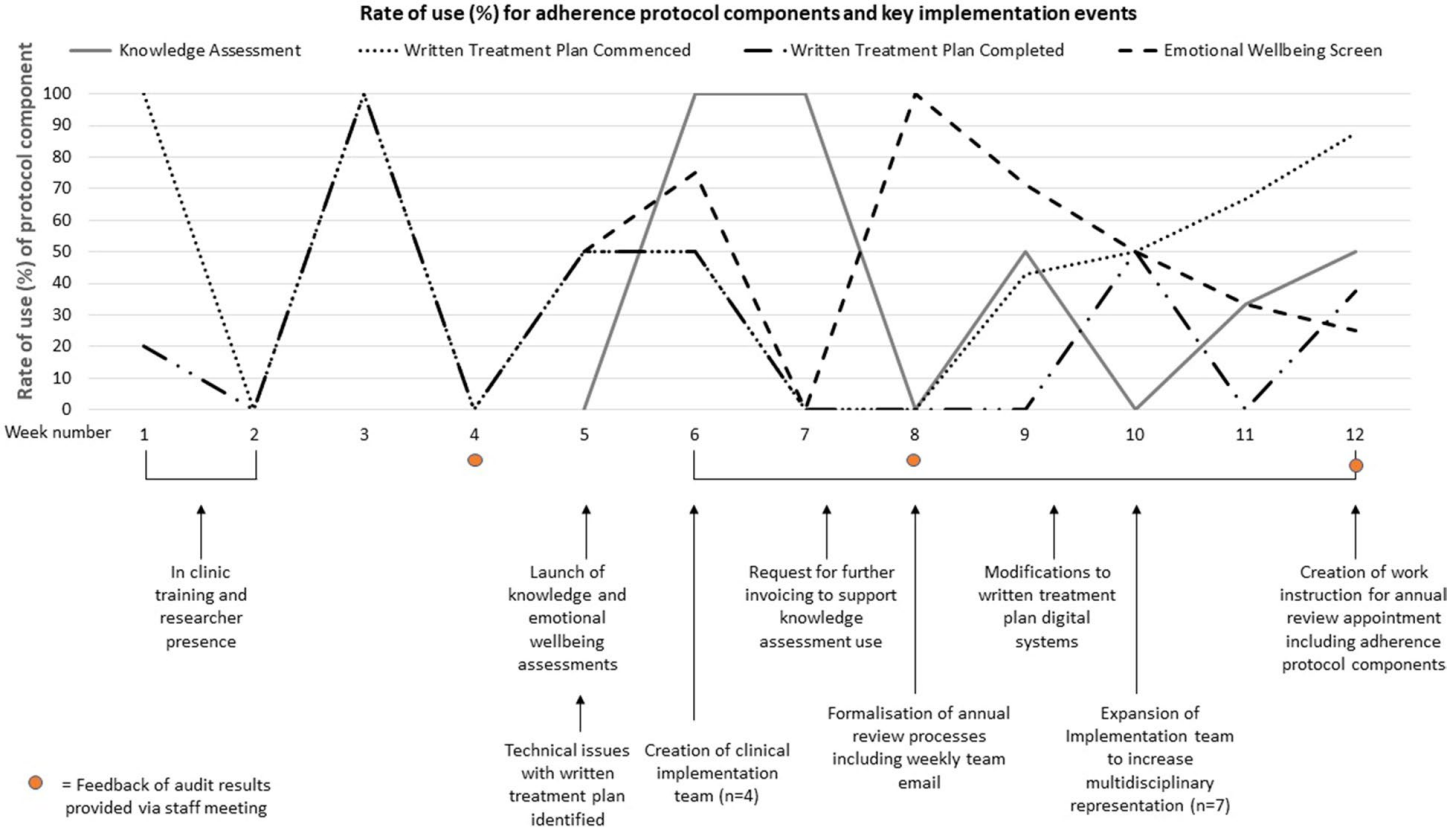
Weekly use of the protocol components and outline of key implementation events

During the three-month implementation phase (April to June 2019), 359 outpatient appointments were completed in the cystic fibrosis clinic. Thirty-five families attended their annual review appointment and medical chart audits demonstrated that the MAP was being used with 43.8% (knowledge assessment) to 57% (mental health screening) of eligible consumers by the third month of implementation. Rates of use over the three-month period are outlined in Fig. 1. As multiple team members were required to complete the written treatment plan, differences were noted between the commencement and completion rates. Commencement rates averaged 65% in the final month of the implementation phase; however, completion rates were lower at 45%. Tailored implementation strategies were introduced in response to the monthly auditing process (Fig. 1).

A key finding of the auditing process was that an insufficient number of consumers were being booked for annual reviews per month. In three months, only 10% of consumers had completed their annual review appointments compared to an anticipated 25%. This resulted in a lower number of eligible recipients for the MAP. Addressing systemic barriers to the booking process became an implementation strategy at the end of the first month. In week 6, an implementation group was created, initially with CF nursing staff (2–3 nurses) that expanded to include a social worker, physiotherapist and dietician. Most of this group had consulted on the design of the MAP. Regular meetings (fortnightly) were introduced to address feasibility and systemic barriers identified (via auditing) to improve fidelity. Annual review rates had improved by 4% in the final implementation month of the trial due to active exploration and problem solving of the annual review processes.

Digital screening assessments^1^ were completed with 135 young people, adolescents or parents. The parent mental health screening was the most utilised tool (*N* = 80). Social workers of the clinic administered the assessments digitally (using iPad). Immediately after completion, the social worker viewed the results and initiated follow up care as clinically appropriate. Screening identified that 41.67% (*n* = 10) of young people (12 years+) and 19% of parents (*n* = 18) assessed were in the “moderate to severe” range for anxiety and/or depression. Of the consumers identified as ‘at risk’, 86% (*n* = 24) received follow up care (such as occupational therapy, social work review or referral to mental health services). The follow up rate was discussed with the clinical team at project completion to raise awareness, identify ongoing barriers to care and determine further modifications required by the clinical team.

The knowledge assessment results identified that none of the children aged below 11 years who received knowledge screening presented with sub-optimal knowledge assessment results (scores below 50%). However, 15% (*n* = 20) of young people aged over 11 years who completed screening were flagged to have sub-optimal knowledge about their condition and treatments. These results were fed back to the team via the occupational therapists to suggest further educational intervention required.

### Acceptability and appropriateness

The MAP components were observed to have high perceived fit (i.e., acceptability) with the CF outpatient clinic on a five-point scale (1 = “Not at all” and 5 = “A lot”) with a median of 5 (IQR: 4,5). Clinicians perceived the tools to be helpful to their work (i.e., appropriate), however greater variation was observed in the responses to this question (Median: 5, IQR: 3,5). Clinicians reported that the tools had a high level of helpfulness for the families of the clinic (Median: 5, IQR: 4,5), however parents had a slightly lower perceived feeling of usefulness regarding the clinic tools (Median 4, IQR:4,5). Survey results highlighted high staff awareness of the clinic communication form and written treatment plan (100%), but less for mental health screening (90%) and knowledge assessment (70%). Qualitative data collected via survey was triangulated with the technical assistance log to identify key factors that impacted implementation during the three-month period (Table 4).

**Table 4:** Post Implementation factors identified by clinician and parent survey and technical assistance logs

CFIR constructs	Factors identified by stakeholders	Valence	Description/ Quote
**Inner Setting** **Implementation climate**	Compatibility	(U)	The pre-implementation co-design and facilitation supported compatibility between the local adherence protocol and local processes. However, modifications to the local adherence protocol continued throughout the implementation phase, into the last week. Clinicians reported that a preference for components to be embedded with existing systems to reduce double handling of information (such as entry into electronic records and written treatment plan) *“Improve by integrating to current system with excel spreadsheet at front desk. Streamline to make more efficient” (Clinician survey, post-implementation)*
**Readiness for intervention**	Available Resources	(−)	Clinicians identified time, available electronic systems, clinic nurse resourcing as barriers to implementation. *“What gets in the way?” “Time pressure of clinic and not even time when in with patients/ families.” (Clinician survey, post-implementation).* *“Any extra work is difficult.” (Clinician survey, post-implementation)*
**Networks and Communication**	Co-ordination	(U)	Completion of the adherence protocol required the physician, physiotherapy, occupational therapist, dietician, nurse, social worker to all review the family within their annual review appointment. Through auditing, it was observed that elements of the protocol were not completed when reviewed in chart audit due to family leaving before being seen by all team members. Clinicians acknowledged that whole team input was impactful on perceived acceptability of tool. *“If [the written treatment plan is] not used consistently with all staff then the efficacy of tool is significantly diminished” (Clinician survey, post-implementation)* It was observed that clinic nurses assumed a coordinator role to support completion by all team members, which positively impacted implementation.
	Formal communication	(−)	Reduced attendance at team meetings impacted diffusion of training information and modifications made to processes. It was a challenge to ensure the awareness of whole team.
**Process** **Engaging**	Getting the whole team on board	(−)	End survey results of clinicians and parents showed varying levels of awareness around adherence protocol components. An implementation team (consisting of nursing, allied health and research team representatives) was formed during implementation phase to support diffusion of information and to support ongoing protocol facilitation.
**Outer Setting** **Needs and Resources of those served by the organisations**	Ability to individualise care	(+)	Clinician acceptability scores consistently suggested that the local adherence protocol components were perceived to be high value for families. *“Very useful and family centred.” (Clinician survey, post-implementation),* *“It’s good for parents to know who needs to see them.” (Clinician survey, post-implementation)* Parents reported that they felt the components were helpful but reported that inconsistent use was a frustration. *“I saw this on e-mail (parent newsletter), if I’m aware that I can use it at clinic that would be great. Didn’t ask me today.” (Parent survey, post-implementation)*
**Characteristics of Individuals**	Individual stage of change/ knowledge	(−)	Clinicians reported that learning new systems, forgetfulness and new habit formation impacted upon individual change. *“Forgetting to use it as it is a new process. Just requires longer use to get used to it”* (Clinician *survey)* Individuals identified gaps in their knowledge and understanding of processes, comments suggest this was linked to ongoing process modifications. *“It’s just sometimes difficult to know where it’s kept (storage of written treatment plan). Needs to be consistent.” (Clinician survey, post-implementation)*
**Intervention Characteristics** **Quality and packaging**	Digital platforms and associated resources	(U)	Unfamiliar technology platforms were introduced to support the requirements of digital screening and treatment plan (electronic access outside of clinic room, multiple authorship and autosave functionality). These digital platforms reduced time and administration associated with use and increased access in and out of the clinic room. Digital systems also required clinicians to use (new) technological systems (Redcap, SharePoint). Additional resources were required to support knowledge assessment use and reduce time impact on clinicians, including creation of “red flag” scores and clinical follow up protocols, as well as feedback and education resources.
**Non- CFIR Domains**	Existing processes	(−)	Inconsistencies were identified within underlying clinic systems. Midway surveys identified that annual review processes were poorly understood by the clinical team. Therefore, pairing the local adherence protocol components with annual review reduced the frequency of use as rate of appointment booking for annual review was lower than anticipated. Inconsistencies were also identified in pre-clinic meeting processes and team communication prior to clinic. Therefore, the CF nurse was unable to inform parents of clinicians planning on seeing them at the clinic via the Clinic Communication Tool.

## Discussion

This is the first study examining the use of a theoretically and conceptually derived implementation strategy, to introduce an adherence protocol into a CF outpatient clinic. The MAP components were used with up to 65% of eligible families at 3 months after introduction. The commencement of a written treatment plan and the use of mental health screening tools were the most successfully implemented components. Both clinicians and caregivers indicated that they considered the MAP to be appropriate and acceptable. There was a high level of agreement among users that the quality of adherence care could be improved by using adherence tools in the clinic. However, despite adaptation and implementation planning, the use of all components by clinicians was inconsistent over the three-month observation period. These findings are congruent with other clinic-based studies that have attempted to integrate an adherence protocol [12, 30, 31].

Clinicians reported that entering adherence information into both the written treatment plan and electronic records increased clinical burden. Clinicians identified that they had insufficient available time to complete protocol components in clinic and reported a perceived training burden when having to learn new tools and processes. The technical logs reveal that components of the MAP were undergoing modifications until the last week of the entire implementation period (3 months). In future, attention should be given to optimising compatibility between adherence tools and the existing clinic systems as this was the most cited barrier to success. It is recommended that teams use pilot testing methods [32] with a small group of patients/ clinicians to facilitate ‘on the ground’ learnings and optimise compatibility before scaling up, thereby reducing the need for clinician retraining and perceived mismatches between the protocol and clinical practice. Alternately, Quittner et al. (2019), suggest that adherence tools may be better suited to sit outside general clinic reviews and suggests telehealth sessions could be offered in addition to the team-based CF review [12].

New findings were uncovered when evaluating the factors that impact standard, clinic-based adherence care. Firstly, *networks and communication.* This factor refers to “the nature and quality of webs of social networks and […] the quality of formal and informal communications within an organization” ([23] p.g. 8). At pre-implementation, both formal and informal communication networks were identified by clinicians as likely to impact implementation. Clinicians reported existing poor staff attendance at the clinic meetings and disjointed communication channels across medical and allied health staff. During the implementation phase, communication networks influenced the diffusion of information about the MAP. Training was delivered to clinicians through existing team meetings, via email and informally (through verbal discussion and demonstration by the research team) during the outpatient CF clinic. Despite these efforts, almost a third of surveyed staff in the post-implementation phase had low awareness of some MAP components (particularly the knowledge assessment). As low attendance at training sessions was also described when introducing the IMPACT Protocol [12], communication strategies to support diffusion of information to the clinicians who are expected to operationalise practice changes warrants further consideration. Use of existing communication mechanisms to reduce time burden on staff is a plausible assumption. However, the communication strategy should also consider the what, who, how, how much and how often information needs to be delivered to enhance diffusion. The introduction of an implementation team (including researchers and clinical champions) was noted to improve communication, particularly the feedback of concerns and suggestions to the research team from local clinicians [32].

Secondly, *underlying clinical processes *(the assumed or outlined process components that support daily clinical practice in health care clinic) including administrative booking and scheduling tasks had significant valence on the reach of the MAP. Despite annual review being considered standard care [33], auditing revealed that the actual number of monthly annual reviews being completed was underestimated. The team’s understanding of annual review and underlying processes were inconsistent. Use of systems analysis in the pre-condition phase of planning would likely have uncovered the low annual review numbers. When adherence protocol processes are designed to be linked with clinic flow processes, it is imperative that these underlying systems are optimised.

Thirdly, *culture* (the “norms, values, and basic assumptions of a given organization” ([23] p.g. 8) was described to play a role in how clinicians engaged in adherence care. The majority of clinicians involved in the pre-implementation focus groups described a belief that “adherence” was an unattainable target and that the idea of families achieving full adherence to their CF treatment regimens was unrealistic. Clinicians stated they were conscious of the significant burden that daily treatments placed on families, and that this impacted how they provided adherence focused care. Clinicians described acting in a protective way to avoid overwhelming the family. For example, the clinician would alter the message that they provide to the family, lowering their expectations about treatment adherence to be “more realistic”; or the clinician would present less treatment options/recommendations according to their perception of the family’s capacity to manage. In contrast, parents/carers reported that they would prefer their team to openly discuss more treatment options with them, rather than assuming the family’s burden. Some parents spoke to a feeling of having to ask for additional treatments that they had read about online or feeling their care team was holding back in their recommendations. Understanding and changing clinician’s beliefs and assumptions about the efficacy of their adherence care appears to impact how care is provided. This is further demonstrated by Casper et al’s study, in which clinicians’ beliefs that patients would inaccurately answer adherence questions impacted the frequency at which they would administer standardised assessment tools [30]. Similarly, Riekart et al. (2015) uncovered that clinicians’ beliefs about the efficacy of their own ability to change behaviour through adherence counselling was a challenge to daily adherence practices [13].

### Limitations

The limitations of this study should be noted. The purposive recruitment of clinicians and families to the study may have introduced respondent bias, as interested parties were more likely to allocate time to engage in the study. Therefore, views of stakeholders with low interest or low investment in adherence service redesign may be under-represented (but impactful on outcomes). Adolescent voices were only represented with two young people (both female) in the study. The inclusion of additional adolescent participants may have given a wider range of experiences including the male adolescent perspective. This study aimed to gather a “whole of clinic” (parents of, and children 0–19 years old) view of standard adherence practices which limited the number of participants (regardless of gender) included in each demographic group. It is recommended that future qualitive studies could explore adherence views targeting more specific demographic groups (e.g. parents of toddler, adolescents) to achieve a broader understanding of that group’s experiences.

Due to the inherent complexities and contextual factors unique to each CF clinic, the results are not generalisable but may be applicable to other health professionals working in CF or chronic disease clinics. The implementation of the problem-solving component of the original protocol was not included in this study to manage the number of new resources and processes introduced at once, to support sustainable change. However, following service evaluation using the key factors outlined in this paper (e.g., systems analysis and local adaption of tools to improve compatibility), the implementation of a collaborative problem-solving intervention is recommended to improve comprehensive adherence care and support a shared care treatment model. Future studies should allow for a longer implementation phase to allow a staggered rollout of the MAP including problem solving training, troubleshooting and booster training.

The level of concern among clinicians and consumers regarding mental health and its impact on adherence was not anticipated at the commencement of this study. Mental health screening was included in the MAP and screening results demonstrated that high anxiety and depression rates were prevalent in just over 40% of young people 12 years and over (*n* = 10), and almost 20% of parents (*n* = 18). Prior to commencement of the study, senior psychology leaders were consulted to develop the care pathways for managing results of mental health screening. However, psychologists were not involved in the implementation teams due to limited availability at the study setting. Young people with mild to moderate depression/anxiety scores were referred to community mental health supports as they did not meet criteria for tertiary hospital-based psychology services. Whilst it was beyond the scope of this implementation study to examine the impact of introducing mental screening on rate of adherence to treatment, future studies would benefit from use of an implementation team including psychology and further investigation of the incidence of mental health concerns in children and young people with CF. Additionally, investigating the efficacy of accessing cystic fibrosis trained, internal psychology resources within the care team compared to generic community services to support unique needs of young people and parents impacted by cystic fibrosis is recommended.

## Conclusions

An adherence protocol is not an “off the shelf” solution to the adherence challenge in CF. Lessons can be learnt from observing and evaluating their implementation within the outpatient setting. The results of this study support that adherence protocols are considered appropriate and acceptable to both clinicians and consumers. However, results suggest that adherence protocols may have limited feasibility in the outpatient hospital setting, despite tailored approaches.

Implementation outcomes were improved with the use of implementation champions with multidisciplinary representation to improve team awareness of service changes; the use of pilot trials and systems analysis to assess compatibility with clinic workflow; and regular audit and feedback by the implementation group members or departmental leaders. To improve the quality of adherence care, further exploration into how clinicians’ beliefs about adherence impact the provision of care is warranted. The inclusion of family preferences in appointment planning and moves towards shared care models may improve efficiency and coordination of adherence care.

## Supplementary Information

**Additional file 1.** Clinician focus group question guide.

**Additional file 2.** Parent/young person interview question guide.

**Additional file 3.** Purpose designed checklists for clinical auditing.

**Additional file 4.** Clinic Communication Form.

## Data Availability

The datasets generated and analysed during the current study may be available if appropriate permissions are obtained (by those seeking to access the data) from the data custodians with appropriate ethical and governance approvals from Children’s Health Queensland Hospital and Health Service Human Research Ethics Committee who can be contacted at the following email: CHQETHICS@health.qld.gov.au. The co-author MS can be contacted at megan.simons@health.qld.gov.au for further information regarding access to the dataset.

## Abbreviations

CF: Cystic Fibrosis
REP: Replicating Effective Programs
STaRI: Standards for reporting implementation studies
MAP: Multicomponent Adherence Protocol

## Footnotes

1 While assessment and screening results were not the primary outcomes of interest in the study, it is important to note that eligibility for the standardized assessment components was dependent on the patient’s age. Only patients aged 12 years and over were eligible for mental health screening and only patients aged over six years were eligible to complete the knowledge assessment [12].
