# Supplementary material for: Introducing a MAP for adherence care in the paediatric cystic fibrosis clinic: a multiple methods implementation study
Source: BMC Health Serv Res. 2022 Jan 26;22:109. doi: 10.1186/s12913-021-07373-5 (PMC8790869; doi:10.1186/s12913-021-07373-5)
Supplement: Supplementary file 1 — Additional file 1. Clinician focus group question guide. [file 12913_2021_7373_MOESM1_ESM.docx]

| 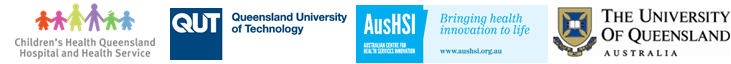 | |
| --- | --- |
| **Topic 1: Current clinic approach** | **How is adherence currently managed in the CF team, from your perspective?**  Prompt questions if needed:  - “I’m interested in the who, what, where, when of current adherence work.”  - Whose role is it to conduct adherence work?  - When does adherence work occur?  - How does adherence work occur? |
| **Topic 2: Individual clinician perspective** | **How would you describe your experience of doing this kind of work in CF?**  Prompt questions if needed:  - How would you describe your feelings of confidence, level of competency, knowledge in delivering adherence work? |
| **Topic 3: Why do adherence work? What is the impact?** | **What is the goal / outcome you are working towards when engaging in adherence work w/ CF families?**  Prompt questions if needed:  - What impact are you aiming to have with your adherence work?  - What do you feel is the outcome of targeting adherence for a family?  - What does adherent patient look like/ what are the behaviours or the goals you are working towards in targeting adherence? |
| **Topic 4: Barriers** | **What are the current challenges in completing adherence work?** |
| **Topic 5: Needs assessment/ areas for improvement** | **What helps you in this team to complete adherence work?**  Prompt questions if needed:  What could the clinic do better to improve adherence outcomes?  Name two things that assist / would assist you to complete adherence work. |
