# Supplementary material for: Introducing a MAP for adherence care in the paediatric cystic fibrosis clinic: a multiple methods implementation study
Source: BMC Health Serv Res. 2022 Jan 26;22:109. doi: 10.1186/s12913-021-07373-5 (PMC8790869; doi:10.1186/s12913-021-07373-5)
Supplement: Supplementary file 2 — Additional file 2. Parent/young person interview question guide. [file 12913_2021_7373_MOESM2_ESM.docx]

| 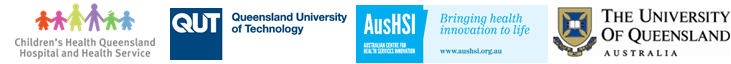 | |
| --- | --- |
| **Topic 1** | **Parent**: So, this interview is really looking at how CF clinic works with families. Can you think of a time when staff at CF clinic have asked about your child’s treatments or if they have tried to help you or your child to improve how they manage their treatments? Can you tell me about your experience of this at the CF clinic?  **Young person**: So, I want to know how CF clinic works with families. Can you think of a time when people at CF clinic have asked you about your treatments or if they have tried to help you to improve how you do your treatments?  Can you tell me about your experience of this at the CF clinic? |
| **Topic 2** | **Parent:** What would you say if I asked you, whether or not clinic staff provide a consistent approach / message around managing or improving your child’s completion of their CF treatments?  **Young person:** I want to know whether or not people at CF clinic give you the same message about doing and improving the way you do your CF treatments, what do you think? |
| **Topic 3** | **Parent** When you are at CF clinic, what do you feel helps you and your child to maintain or improve your child’s completion of their CF treatments?  **Young person:** When you are at CF clinic, what helps you to think about doing or improving how you do CF treatments? |
| **Topic 4** | **Parent** When you are at CF clinic, what do you think are the barriers to addressing or improving treatment completion?  **Young person:**  When you are at CF clinic, what makes it difficult to think about doing or improving the way you do your CF treatments? |
| **Topic 5** | **Parent:** At CF clinic, what has been your experience receiving education about CF, CF treatments and treatment skills such as medication dosing, equipment cleaning, therapy techniques?  **Young Person:** Tell me what it is like at CF clinic when you are told about what CF is, CF treatments and treatment skills such as medication dosing, equipment cleaning and therapy techniques? |
| **Topic 6** | **Parent:** How would you like to be supported by the CF clinic to help manage your child’s daily CF treatments?  **Young Person:** What do you think the CF clinic could do to help you to manage your daily CF treatments? |
