# Supplementary material for: Introducing a MAP for adherence care in the paediatric cystic fibrosis clinic: a multiple methods implementation study
Source: BMC Health Serv Res. 2022 Jan 26;22:109. doi: 10.1186/s12913-021-07373-5 (PMC8790869; doi:10.1186/s12913-021-07373-5)
Supplement: Supplementary file 3 — Additional file 3. Purpose designed checklists for clinical auditing. [file 12913_2021_7373_MOESM3_ESM.docx]

| 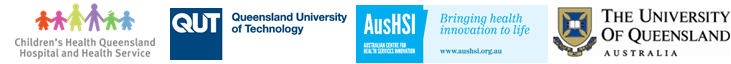 | | |
| --- | --- | --- |
| **Week __________ Date _____________** | | |
| **Item** | **Key Components** | **Number /3** |
| **Annual Reviews** | **/ week** |  |
| Knowledge assessment | - Knowledge assessment was not completed (0) - Knowledge Assessment was completed within annual review visit (1) - Results were documented in iEMR (included total score for Nutrition, Lung Health, CF Health, Treatments) (2) |  |
| My CF Action Plan  Goals | - No CF plan (0) - A partially completed action plan is saved on H drive (1) – an action plan was commenced but not all treatments recorded. - A completed Action plan is saved on H drive (2) - A completed Action plan is saved on H drive and scanned into iEMR (3) - Therapist has documented the [provision of a written treatment plan to family] - 0 patient goals were made (0) - 1-3 patient goals are documented (1) - >3 are recorded goals are documented (2) - Goal appears collaborative from patient input – record examples – subjective - comments |  |
| Emotional wellbeing screening | - Emotional Wellbeing assessment was completed (parent) (1) - Emotional wellbeing assessment was completed (child) – if older than 12 y.o. (1) - Results are documented in iEMR (1) |  |
