# Supplementary material for: Introducing a MAP for adherence care in the paediatric cystic fibrosis clinic: a multiple methods implementation study
Source: BMC Health Serv Res. 2022 Jan 26;22:109. doi: 10.1186/s12913-021-07373-5 (PMC8790869; doi:10.1186/s12913-021-07373-5)
Supplement: Supplementary file 4 — Additional file 4. Clinic Communication Form. [file 12913_2021_7373_MOESM4_ESM.docx]

Additional File 4: Clinic Communication Form

| 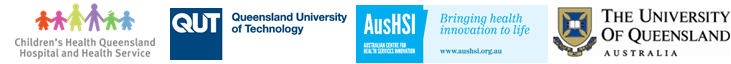 | | | |
| --- | --- | --- | --- |
| **Cystic Fibrosis Clinic** | | | |
| **Initials: _______________________** | | | |
| **My Clinic Appointment Today** | | | |
| **Completed** | **Team Member** | **Planned** | **Family Requested to See** |
|  | **Lung Function** |  |  |
|  | **Doctor** |  |  |
|  | **CF Nurse** |  |  |
|  | **Physiotherapist** |  |  |
|  | **Dietician** |  |  |
|  | **Occupational Therapist** |  |  |
|  | **Social Worker** |  |  |
|  | **Research** |  |  |


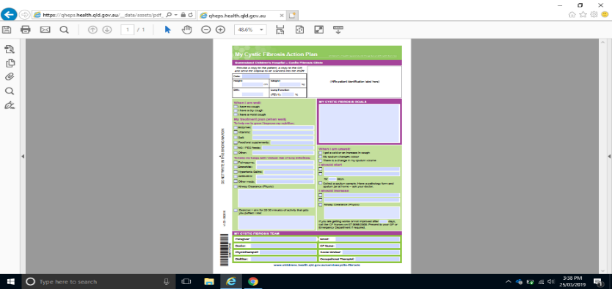


Is a written CF Action Plan required today? (Annual review Part A + B)

*Ensure a printed copy been provided to the family before they leave clinic.*

**Notes**
